# The effect of a finishing diet supplemented with γ-aminobutyric acids on carcass characteristics and meat quality of Hanwoo steers

**DOI:** 10.5713/ajas.20.0224

**Published:** 2020-08-21

**Authors:** Farouq Heidar Barido, Chang Woo Lee, Yeon Soo Park, Do Yeong Kim, Sung Ki Lee

**Affiliations:** 1 Department of Applied Animal Science, College of Animal Life Sciences, Kangwon National University, Chuncheon 24341, Korea; 2 Gangwon Province Livestock Research Institute, Hoengseong 25266, Korea

**Keywords:** γ-Aminobutyric Acid (GABA), Supplementation, Meat Quality, Hanwoo Steers

## Abstract

**Objective:**

This study was conducted to investigate the effects of supplementation with rumen-protected γ-aminobutyric acid (GABA) on carcass characteristics and meat quality of Hanwoo steers.

**Methods:**

Eighteen Hanwoo steers with an average initial weight of 644.83±12.91 kg were randomly allocated into three different groups. Each group consisted of 6 animals that were treated with different diets formulated based on the animals’ body weights. The control (C) group was fed a basal diet consisting of concentrate and rice straw with 74% total digestible nutrients (TDNs) and 12% crude protein (CP). The two other groups were treatment groups; one group was fed a basal diet (74% TDNs and 12% CP) supplemented with rumen-protected GABA at a dose of 150 mg/kg feed, and the other group was fed a basal diet (74% TDNs and 12% CP) supplemented with GABA at a dose of 300 mg/kg feed.

**Results:**

The GABA supplementation significantly contributed to better growth performance (p<0.05), especially the weight gain and average daily gain. It also contributed to the lower cooking loss (p<0.05), improvements in essential antioxidant enzymes and stable regulation of antioxidant activities in the *longissimus lumborum* of Hanwoo steers, as represented by the lower formation of malondialdehyde content within the meat, the inhibition of myoglobin oxidation indicated by the retention of the oxymyoglobin percentage, and the suppression of metmyoglobin percentage during cold storage (p<0.05).

**Conclusion:**

Higher doses of GABA may not significantly promote better animal performance and meat quality, suggesting that dietary supplementation with GABA at a dose of 100 ppm is sufficient to improve the meat quality of Hanwoo steers.

## INTRODUCTION

Global warming is leading to an increase in earth’s surface temperature. During the 20th century, records show that earth’s surface temperature increased by 0.6°C, which increased the occurrence of heat shock [1]. It is widely known that heat shock due to increasing ambient temperatures has significant detrimental effects on animal performance, ultimately affecting meat quality [2]. One study [3] revealed that heat stress could have detrimental effects on meat quality through two mechanisms: direct effects of continuous exposure to heat stress on organ and muscle metabolism, which can lead to the formation of pale, soft, oxidative meat in poultry and dark, dry meat in beef and may even persist post slaughter, and maintenance changes in response to ambient temperature changes that indirectly affect meat quality. Worse yet, heat stress could alter important economic traits in the beef industry, leading to increased maintenance requirements and weight loss, even during distribution [4]. Heat stress induces weight loss through interference with the hormonal system, such as thyroid hormone. This hormone functions to mitigate metabolic heat and maintain the water and salt balance that ultimately regulates body homeostasis. Continuous disturbance of thyroid hormone by heat exposure can affect dry matter intake, cause metabolism imbalances and adversely affect weight gain during maintenance [5].

To overcome the negative effects of heat stress, especially metabolic imbalances, supplementation with protein either with essential amino acids, non-essential amino acids or even nonprotein amino acids is widely utilized [6]. Numerous studies have proven that dietary amino acid supplementation in Hanwoo beef cattle is able to increase meat quality through the inhibition of malondialdehyde (MDA) content and improvement in antioxidative enzymes during storage, resulting in a better antioxidative profile, improved meat appearance with a redder color and stronger water-holding capacity (WHC), and a more tender texture [7]. Therefore, there is increasing research interest regarding the utilization of amino acids as dietary supplements to overcome animal stress during heat exposure [8].

γ-Aminobutyric acid (GABA) is a nonprotein amino acid composed of four carbons that is synthesized through a complex mechanism involving an irreversible α-decarboxylation reaction of glutamic acid via catalysis of glutamic acid decarboxylase enzyme with pyridoxal phosphate (the active form of vitamin B_6_) as a cofactor [9]. GABA plays many important roles in living organisms, such as its critical role in the Krebs cycle in plants and microorganisms and its function as an inhibitory neurotransmitter in the brains of vertebrates [10]. GABA is considered a bioactive compound with numerous functional effects having behavioral and physiological outcomes; furthermore, it plays an important role in mammalian central thermoregulation [11,12]. Numerous studies have been conducted to determine the benefits of GABA supplementation for animal performance and productivity during heat stress. GABA supplementation increased the performance of chicks during heat exposure by mitigating the detrimental effects of heat stress [13]. A probiotic made from a GABA-producing organism also significantly improved the laying performance and productivity of hens under heat stress [14] by strengthening antioxidative enzymes and increasing feed intake under heat stress conditions [15]. In ruminant animals, it has been well documented that supplementation of rumen-protected GABA is useful in helping early lactating cows maintain feed intake, lactation performance and animal health in response to heat stress [16]. To the best of our knowledge, few studies have examined the effects of supplementation with rumen-protected GABA on meat-producing animals, particularly beef cattle. Furthermore, limited information exists regarding the effects of supplementation in one valuable cattle breed in Korea (*Bos taurus coreanea*). Therefore, the aim of this study was to investigate the effect of dietary GABA supplementation on carcass characteristics and meat quality of Hanwoo steers.

## MATERIALS AND METHODS

### Animal care

Animal handling considering the animal welfare was performed under the guidelines from the Institutional Animal Care and Use Committee (IACUC) of the Gangwon Province Livestock Research Institute (KNU IACUC-2017-05).

### Animals, diets and feeding

This study was conducted at the feedlot farm of Gangwon Province Livestock Research Institute, utilizing eighteen Hanwoo steers with an average initial weight of 644.83± 12.91 kg. Cattle were randomly allocated into three different groups and placed in three distict pens with a size of 5×10 m each. Each pen group consisted of 6 animals that were fed for 120 days with different diets formulated based on the animals’ body weights. The diet formulation was based on the recommendations from the published standard guidelines [17]. Daily feed intake was discriminated into control and treatments; the control (C) group was fed a basal diet consisting of rice straw (1.5 kg per day) and concentrate (10 kg per day), which contained 74% total digestible nutrients (TDNs) and 12% crude protein (CP). The two other groups were treatment groups; treatment 1 group was fed with control diet supplemented with rumen-protected GABA at a dose of 150 mg/kg feed per day, while for treatment 2 received control diet supplemented with GABA at a dose of 300 mg/kg feed. Daily feed and access to water was given as *ad libitum* and the rumen-protected GABA supplement was administered as a top-dressing powder over 120 days.

### Growth performance

Individual body weight was recorded at the beginning and after 120 days of feeding. Total weight gain and average daily gain (ADG) were calculated.

### Carcass traits and sampling

After the 120 days of the finishing period, the animals were sacrificed to measure the effects of the treatment diets on carcass traits. Carcass weight, yield grade (hot carcass weight, backfat thickness, and ribeye area) and quality grade (marbling score, meat color, fat color, firmness, and maturity) were evaluated based on the Korean carcass grading system. The strip loin (*longissimus lumborum*) of each animal was cut and packed in polyethylene zipper bags at 24 h postmortem and subsequently transported to the laboratory. Samples were stored in a chilling room at 2°C±2°C overnight. The strip loin from one animal was sliced into six steaks (2 cm thick). To observe the effect of storage on color, pH, lipid oxidation and freshness, three steaks from each animal were placed on Styrofoam, wrapped with oxygen-permeable film and stored at 5°C±0.5°C in the dark for 3, 6, and 9 days. Two other steaks from each animal were used for cooking loss and shear force analyses, and the remaining steaks were used to analyze color, pH, lipid oxidation, and freshness at day 0 of storage. The analyses of proximate composition, WHC and fatty acid composition were also performed using day 0 samples on day 2 postmortem. Samples used for fatty acid analysis were stored at −24°C under vacuum and thawed overnight prior to analysis.

### Proximate composition

Samples were ground using a food grinder (HMF-1600 PB, Hanil Electric, Seoul, Korea) at medium speed for 10 s. Proximate composition was determined according to method of the Association of Official Analytical Chemists (AOAC) [18]. The percentage of moisture was determined by drying the samples in the oven at 105°C for 24 h, while analysis of the crude fat content was conducted with ether using the Soxhlet solvent system. Nitrogen content was observed using the Kjeltec system (2200 Kjeltec Auto Distillation Unit, Foss, Hilleroed, Sweden), and CP was calculated as the nitrogen content multiplied by 6.25. Crude ash was determined by burning the samples in the muffle furnace at 550°C for 12 h.

### Instrumental surface color

The surface meat color was measured immediately after the blooming phase was completely achieved (1 h after slicing). A colorimeter (CR-400 Konica Minolta Sensing Inc., Tokyo, Japan) was used to record each sample’s lightness (L*), redness (a*), and yellowness (b*). The 8 mm aperture of the source of illuminant light C (2° observer) was calibrated using a white plate (Y = 93.6, X = 0.3134, and y = 0.3194). Measurements were performed 5 times for each sample.

### Water-holding capacity, cooking loss and shear force

The WHC was measured according to a previously published method [19] with slight modifications. Briefly, 5 grams of each ground sample was transferred into a centrifuge tube, and the tube was sealed and then subjected to heating in a water bath for 30 min at 75°C. The tube was then cooled in flowing cold water for 30 min and subsequently centrifuged at 980 *g* for 10 min at 24°C (1248R, Labogene, Lynge, Hovedstaden, Denmark). The supernatant was then decanted and measured, and the moisture content of both the raw meat and the supernatant was determined using the AOAC method.

Cooking loss is defined as weight loss after heating until a medium degree of doneness is achieved (68°C to 72°C internal temperature). Briefly, samples were weighed to get an initial weight (W1) of 70±5 g. Samples in duplicate were then placed in the polyethylene zipper bags and cooked by water bath immersion at 80°C. The cooked samples were subsequently removed from the bags, cooled at 2°C±2°C for 30 min and weighed (W2). The percentage of cooking loss was obtained by comparing weight loss (W1W2) against W1.

The cooking loss samples were subsequently used to measure the tenderness (toughness) of the meat by performing the Warner-Bratzler shear force test using TA-XT2*i* Plus (Stable Micro Systems, Surrey, UK). In accordance with previously published methods [20], the meat was cut into 1.5 cm×1 cm samples and then positioned under the V blade, where it was cut through the strand with constant speed through the gap of the instrument’s table (assay parameters: pretest speed, 2.0 mm/s; test speed, 1.0 mm/s; and posttest speed, 10 mm/s). Each sample was repeated eight times.

### Antioxidant enzyme activity

Superoxide dismutase (SOD), catalase (CAT) and glutathione peroxidase (GSH-Px) were measured using previously published methods [20] in aqueous meat extracts dissolved in 50 mM phosphate buffer (pH 7.0 at 25°C). The decrease in CAT activity during storage was also calculated according to established methods [20]. Briefly, 100 μL of filtered supernatant was mixed with 2.9 mL of 30 mM H_2_O_2_ within a crystal cuvette (light path, 1 cm). The decrease in absorbance at 240 nm was recorded every 10 s for 2 min, and the CAT enzyme was recorded as units/g sample. Subsequently, the determination of SOD during storage was measured based on the pyrogallol autoxidation method, as explained previously [20], with slight modification. Briefly, a mixture consisting of 50 μL of filtered supernatant, 3.025 mL of 50 mM Tris-cacodylate-diethylenetriaminepentaacetic acid buffer (pH 8.2 at 25°C) and 50 μL of 24.8 mM pyrogallol was measured at 420 nm every 15 s for 2 min against a blank. The expression of one unit of SOD reflects the amount of SOD required for 50% pyrogallol autooxidation in the meat extract at pH 8.2 and 25°C, while the GSH-Px activity measurement was performed using the well-documented method of Flohé and Günzler [21] with slight modification. Briefly, a mixture consisting of 0.5 mL of phosphate buffer, 0.001 M of ethylene diamine tetra acetic acid (EDTA), 0.1 M NaN3, 100 μL of the assay mixture containing 5 units mL-1 glutathione reductase in 50 mM phosphate buffer, 100 μL of 10 mM L-reduced glutathione, 100 μL of 1.5 mM nicotinamide adenine dinucleotide phosphate in NaHCO_3_, and 100 μL of 1.5 mM H_2_O_2_ within a 10-mm precision cell (104-QS, Hellma Analytics, Müllheim, Germany) were mixed with 100 μL of filtered supernatant. The mixture was subsequently incubated for 5 min, and the GSH-Px activity was documented by recording the decrease in absorbance of the incubated mixture at 340 nm over 2 min. GSH-Px activity was expressed as units g-1 sample.

### pH and lipid oxidation rate

The pH of the samples was measured using previously published methods [22], in which a 5 g sample was added to 45 mL of distilled water and subsequently mixed with a homogenizer (PH91, SMT Co., Ltd., Chiba, Japan) at 10,000 rpm for 60 seconds. The pH of the well-homogenized meat was obtained using a pH meter; calibration of the pH meter was performed using technical buffer solutions consisting of acid (pH 4.01), alkali (pH 9.00), and neutral (pH 7.00) components with an automatic temperature compensation program (Seven Easy pH, Mettler-Toledo GmbH, Greifensee, Switzerland).

Measurements of lipid oxidation were performed using 2-thiobarbituric acid-reactive substances (TBARS). A 0.5 gram sample was placed in a 25-mL TBARS test tube and was prepared in three repetitions; 0.1 g of antioxidant mixture (consisting of 54% propylene glycol, 40% Tween 20, 3% butylated hydroxytoluene, and 3% butylated hydroxyanisole) was transferred into the tube, after which 3 mL of 1% thiobarbituric acid in 0.3% NaOH was added. Immediately after vortexing, 17 mL of 2.5% trichloroacetic acid in 36 mM HCl was added, and the tube was closed. The sample was then subjected to heating in a water bath (BW-20G, Biotechnical Services, Inc., North Little Rock, AR, USA) at 100°C for 30 min. The tube was immersed in cold water for another 15 min. An aqueous sample (5 mL) was transferred into a new 15 mL centrifuge tube and mixed with 3 mL of chloroform. The mixture was then centrifuged at 2,400×g for 30 min at 4°C (1248R, Labogene, Lynge, Denmark) to separate out impurities. The absorbance was measured at 532 nm by using a UV spectrophotometer (UV-mini 1240 PC, Shimadzu Corp., Kyoto, Japan) against a blank containing only distilled water. Each sample was analyzed three times, and data were expressed in the form of mg MDA/kg sample.

### Myoglobin content

One gram of beef sample was homogenized with 8 mL of sodium phosphate solution (0.04 mol/L, pH 7) and subsequently centrifuged at 10,000 g for 15 minutes. The resulting supernatant was filtered through Whatman filter paper, and the extract absorbance was read at 572, 565, 545, and 525 nm against a blank using a spectrophotometer (iMark680, Bio-Rad, Hercules, CA, USA). The proportions of the DMb, OMb, and MMb were then calculated.

### Fatty acid composition

The determination of fatty acid composition was performed using an Agilent gas chromatography system (6890N; Agilent Technologies, Santa Clara, CA, USA) with an automatic sampler (7683; Agilent Technologies, USA). Meat fat was extracted with a chloroform-methanol (2:1 v/v) solution according to a previously published method [22], and the samples were prepared in duplicate. Using another published method [18], the fatty acids were converted into methyl esters and subsequently dissolved in 1.5 mL of hexane. A 1 μL sample was then injected into the gas chromatograph port by the automatic sampler using the following injector settings: 250°C with a split ratio of 100:1. Separation of the fatty acid methyl esters was performed using a WCOT-fused silica capillary column (100 m×0.25 mm i.d., 0.20 μm film thickness; Varian Inc., Palo Alto, CA, USA) with 1.0 mL/min helium flow at the following oven settings: 150°C/1 min, 150°C to 200°C at 7°C/min, 200°C/5 min, 200°C to 250°C at 5°C/min, and 250°C/10 min. The temperature of the detector was 275°C. Fatty acid identification was performed by comparing each sample’s peak with the retention time of the fatty acid standards (47015-U; Sigma-Aldrich Corp., LLC., St. Louis, MO, USA). Data on peak area of each identified fatty acid were used to obtain the proportion (%) of each against the total identified peak area.

### Statistical analysis

The determination of significance for the treatment groups (pH, TBARS, enzyme activities, color, and myoglobin) during the storage days was performed by using a linear mixed model of two-way analysis of variance (ANOVA). To determine the significant differences in animal performance, carcass traits, proximate content, WHC, cooking loss, shear force, and fatty acid composition, the results were compared with those of the control samples by performing one-way ANOVA. Analyses were conducted using R version 3.6.1 with the “Agricolae” library (The R Foundation for Statistical Computing, Vienna, Austria).

## RESULTS

### Animal performance

The effects of rumen-protected GABA on animal performance are displayed in Table 1. Statistical significance was found in terms of weight gain (p<0.05) and ADG (p<0.05). The group of Hanwoo steers supplemented with rumen-protected GABA possessed a significantly higher weight gain value than the group fed only the basal diet (control). However, no differences were found between the 100 ppm group and the 200 ppm group. Weight gain in the control group, the 100 ppm group and the 200 ppm group was 63.7, 103.1, and 91.9 kg, respectively. Similar results were also obtained for ADG, where Hanwoo steers group supplemented with rumen-protected GABA had a significantly higher ADG than the group fed only the basal diet (control), while there were no significant differences between the 100 ppm and 200 ppm groups. The ADG for the control, 100 ppm and 200 ppm groups was 0.65, 0.86, and 0.77 kg, respectively.

### Carcass traits

Table 2 shows the effects of rumen-protected GABA supplementation on the carcass trait profiles of Hanwoo steers; no significant differences in carcass trait profiles were found after supplementation with rumen-protected GABA (p>0.05). Diets supplemented with rumen-protected GABA did not have a significant effect on either the quantity or quality of Hanwoo steer carcasses.

### Meat quality

#### Proximate composition

The effects of rumen-protected GABA on meat quality are illustrated in Table 3. The cattle group supplemented with rumen-protected GABA generated a higher meat protein value (p<0.05) than the cattle group fed only the basal diet (control). However, the percentage of protein did not differ between the 100 ppm and 200 ppm groups. Protein percentages for the control, 100 ppm and 200 ppm groups were 16.27%, 18.28%, and 19.19%, respectively, while the percentages of moisture, crude fat and ash were not affected by supplementation.

#### Water-holding capacity, cooking loss, and shear force

Supplementation of cattle feed with rumen-protected GABA exerted positive effects on meat quality through an increase in the WHC and lower cooking loss. As shown in Table 3, the group with the highest supplementation (200 ppm) had a significantly higher percent WHC (p<0.05) than the control group. The WHC percentages of the control group, the 100 ppm group and the 200 ppm group were recorded as 74.86%, 75.38%, and 81.31%, respectively. Cooking loss was also significantly decreased (p<0.05) in the group supplemented with 200 ppm of GABA. Cooking loss was found to be 29.65%, 29.01%, and 25.52% for the control, 100 ppm and 200 ppm groups, respectively. Supplementation did not affect meat texture, however, as no significant differences were obtained (p>0.05) for shear force.

### Antioxidant enzyme activity

Table 4 documented the changes of antioxidant enzyme activities during refrigerated storage of Hanwoo meat supplemented with rumen-protected GABA. The activities of CAT enzyme on the initial day was ranging from 321.5 to 350.1 unit/g meat. Its activities was then significantly declined after storage day 6 regardless of treatment groups. The same declining trend was also observed for the SOD enzyme. At the beginning of the storage period, the concentration of SOD ranged from 50 to 52.4 unit/g meat. The concentration was then significantly decreased after day 9 accounted for 43.2 to 51 unit/g meat. While for GSH-Px enzyme activities, it was ranging from 1.51 to 1.59 unit/g meat at the initial day and significantly decreased after storage day 3 with a concentration ranged from 1.05 to 1.21 unit/g meat. Supplementation of Hanwoo steers with rumen-protected GABA did significantly affect the activities of CAT and SOD enzyme (p<0.05). As seen in Table 4, the treatment group with 200 ppm supplementation displayed a higher concentration of CAT enzyme compared to that of the control. However, a limited effect on CAT enzyme was shown in treatment group with 100 ppm supplementation. A similar trend was also obtained for SOD enzyme activities. Regardless of the concentration, treatment groups significantly contributed to a higher level of SOD enzyme compared to the control group since the initial storage period (p<0.05). However, this study did not obtain a significant difference in GSH-Px enzyme activities after supplementation with rumen-protected GABA (p>0.05).

### pH and lipid oxidation rate

The storage experiment was conducted with the intention to observe the changes in meat quality, especially the lipid oxidation rate, color changes, the generation of metmyoglobin and pH changes during cold storage at 2°C±2°C. The results of the storage experiment of *longissimus lumborum* taken from Hanwoo steers after supplementation with rumen-protected GABA are shown in Table 5. The rate of lipid oxidation documented through the TBARS assay is expressed as mg MDA/kg meat. The treatment groups could apparently inhibit the generation of lipid oxidation during cold storage, as demonstrated by a significantly lower MDA content than in the control (p<0.05) from day 3 that was maintained until the final storage day. However, the rate of lipid oxidation in the higher supplementation group (200 ppm) did not differ from that in the 100 ppm group, which implies that supplementation could contribute to the maintenance of meat quality during cold storage. In addition, no interaction was indicated by the analysis of variance between treatment and storage day (p>0.05). While there was no notable difference found between the control and treatment groups from the initial storage day until the end of day 9 with respect to the pH value, which is illustrated in Table 5, a significant increase in the pH value (p<0.001) was recorded in all samples during cold storage.

### Instrumental surface color

Color changes during cold storage were recorded using Commission Internationale de l’Eclairage (CIE) lightness (L*), redness (a*), and yellowness (b*) values, which are shown in Table 6. The CIE lightness value (L*) of all samples did not change significantly across storage days (p>0.05). The CIE redness value (a*), which indicates discoloration of the meat, did differ between the control and treatment groups on day 9 of storage (p<0.001). Both supplement groups (100 and 200 ppm) retained better redness values through the final storage day compared to those of the control group; however, no difference was found between the 100 ppm and 200 ppm supplementation groups. Cold storage significantly lowered the yellowness (b*) of the meat (p<0.001), with no significant differences between the samples.

### Myoglobin content

Determination of myoglobin profiles during storage is important to understanding the changes in oxymyoglobin, deoxymyoglobin, and metmyoglobin. These changes are related to the appearance of the meat since oxymyoglobin is related to the red color, deoxymyoglobin is related to purple color formation and metmyoglobin is related to the deterioration in the color of the meat. Table 6 shows the formation of myoglobin profiles during storage. Myoglobin profiles, especially oxymyoglobin, deoxymyoglobin and metmyoglobin profiles, were significantly affected by the storage period (p<0.001). For the percentage of oxymyoglobin during cold storage, there was a statistically significant difference (p<0.05) between the control and treatment groups, where the treatment groups possessed a higher percentage of oxymyoglobin from day 3 until day 9. However, the 100 ppm treatment group did not differ from the 200 ppm treatment group. Similar results were also obtained for the percentage of metmyoglobin. The formation of metmyoglobin is related to meat discoloration, where a higher percentage indicates a higher rate of meat discoloration. As shown in Table 6, there was an inconsistent pattern for the metmyoglobin formation after supplementation. On day 3, the percentage of metmyoglobin formation was higher in the control group than in the treatment groups, with 38.47%, 32.84%, and 32.17% for the control, 100 ppm, and 200 ppm groups, respectively. There was no difference documented on day 6 of storage. The formation of metmyoglobin at the final storage day was significantly affected by supplementation with GABA, which contributed to the inhibition of metmyoglobin formation and positively contributed to the retention of oxymyoglobin percentage during cold storage. In addition, the higher dose of GABA did not contribute to significantly better myoglobin profiles during storage.

### Fatty acid composition

A basal diet supplemented with GABA had a limited effect on the intramuscular fatty acid composition of Hanwoo steers, as shown in Table 7. Significant differences (p<0.05) were observed only for the myristic acid (C14:0) and eicosapentaenoic acid (C20:5n3) contents, where the treatment groups had a higher percentage of these fatty acid chains than the control group (p<0.05). Additionally, supplementation with GABA seemed to have a significant effect on the percentage of saturated fatty acids. As shown in Table 7, treatment groups had lower levels of saturated fatty acids than the control group, although no difference was found between the 100 ppm and 200 ppm supplementation groups. In addition, monounsaturated fatty acid (MUFA) and polyunsaturated fatty acid (PUFA) levels did not differ between the groups. The MUFAs was dominated by oleic acid (C18:1n9) and palmitoleic acid (C16:1n7) respectively. While the PUFAs was dominated by linoleic acid (C18:2n6), alpha-linoleic acid (C18:3n3), arachidonic acid (C20:4n6) and eicosapentaenoic acid (C20:5n3) respectively.

## DISCUSSION

Heat stress is a main factor causing detrimental effects on animal performance, including meat quality [23]. It related to a complex mechanism of heat stress in altering hormones and enzymes mechanism. As a consequences, it decreased nutrition metabolism through adaptation of neuroendocrine system [13], where heat stress could stimulate the secretion of corticosterone and cathecolamines; hormones associated to a stress condition that contribute to an intiation of lipid peroxidation and destruction of cell membranes, including T and B lymphocytes [24]. Also, depressed mechanism of digestive enzymes; amylase, lipase, trypsin that may impair the effectiveness of nutrients digestibility [13]. Moreover, many studies have suggested that heat stress could decrease dry matter intake during maintenance, which subsequently contributes to weight loss [25]. Ruminant animals, especially cattle, cannot tolerate high ambient temperatures since they mitigate body heat during heat stress through only cutaneous evaporation with the involvement of sweat glands and skin compartments [26]. In the other hand, as a main inhibitory neurotransmitter in central nervous system, GABA plays important role in the action to counteract detrimental effect caused by stress [27]. It is well established that the GABA could elevate the synthesis of growth hormone [27], lower the level of cortisol and stress related enzyme [28] and increase the neuropeptide Y; an amino-acid neuropeptide that serve to reduce the anxiety, stress and a regulation of feed intake and fat storage [29]. A study by Brambilla et al [30] inferred that a reduced activity of GABA in central nervous system during stress could lead to a numerous declined in performance and behavior of animal. Therefore, this study was performed during the summer period for 120 days to understand the effects of GABA supplementation against heat stress in Hanwoo steers. Positively, GABA supplementation of the diet during the maintenance of Hanwoo steers could contributed to a significant increase in weight gain and subsequently to the average daily weight gain. Weight gain in the control, 100 ppm and 200 ppm groups was 63.7, 103.1, and 91.9 kg, respectively. As many studies have reported that high ambient temperatures can impair dry matter intake and ultimately the ADG of the cattle [30], supplementation with GABA could alternatively overcome the detrimental effects of heat stress through increases in digestive enzyme activity, the electrolyte balance and antioxidative enzyme activity, which assist in the mitigation of body heat and the maintenance of feed intake [14]. These results correspond with previous findings [5] in which dietary GABA was shown to increase the dry matter digestibility and therefore increase the ADG in Jinjiang yellow cattle. Higher doses of GABA supplementation did not appear to be effective at improving weight gain and ADG.

GABA serves important functions in many organisms [31]. It is involved in complex protective mechanisms to counteract stress in plants and microorganisms, and it serves as an inhibitory neurotransmitter in several routes within the central nervous systems of animals [32]. The inhibitory effects of GABA from specific binding mechanisms of GABAergic receptors regulate a relaxation response within the body [33]. Therefore, diet supplementation with rumen-protected GABA was expected to provide a relaxed condition within the animals’ bodies, which could ultimately have positive effects on animal production traits. However, this study did not find any significant effect of GABA supplementation on any of the expected traits related to carcass quality (p>0.05). Amino acid supplementation apparently showed an inconsistent pattern of improvement, implying a weak contribution to carcass quality [7]; these findings correspond with those of a previous study [5] in which GABA supplementation had a limited effect on the carcass traits of Jinjiang yellow cattle.

Significant differences were found with respect to meat protein percentage (p<0.05), as the supplementation of rumen-protected GABA seemed to promote a higher rate of protein synthesis. This increase may be due to an important role of GABA in improving nutrition utilization efficiency [13]. In addition, it also helps improve the activity of digestive enzymes such as amylase, lipase and trypsin, which could therefore exert a positive effect on protein synthesis. This finding also corresponds to those of a previous study [34] in which supplementation of rumen-protected GABA significantly improved the apparent digestibility of CP, crude fiber, and calcium contents as a result of improved gastrointestinal sensitivity to physical and chemical stimuli, which promoted more efficient absorption of nutrients and increased feed intake.

The water content of meat is an essential quality parameter considered by the meat processor. Due to its correlation with the final processed meat yield and especially cooking loss, it can have profound economic implications [7]. The diet supplemented with GABA in this study seemed capable of improving meat quality through a decrease in cooking loss percentage and improvements in the WHC. These improvements may have resulted due to a promotive effect of GABA on maintaining the electrolyte balance within the body, which is directly associated with strengthened meat muscle fibers that retain more water during the processing period [14].

To measure the rate of lipid oxidation, this study performed TBARS analysis to record the accumulation of MDA during storage. The MDA content did not differ among the samples, but after day 3, the treatment groups showed lower lipid oxidation rates with a lower MDA content than the control group (p<0.05), which persisted until day 9. Numerous studies have reported that GABA is positively correlated with increased activities of antioxidative enzymes [5]. GABA supplementation could positively affect the regulation of glutamate levels, which is an important raw material for synthesizing GSH-Px, an important enzyme that is closely involved in oxidation mechanisms by protecting the structure of cell membranes from the damage caused by reactive oxidative species [14]. It is also possibly due to an increase in SOD enzyme, which is an active substance derived from cells that scavenges oxygen free radicals [34]. These enzymes are essential antioxidant enzymes that suppress the generation of corticosterone and catecholamines, which play a key role in the overproduction of the oxygen free radicals OH and O_2_. These results are similar to those of previous studies [5] in cattle beef and dairy cows [35] during heat stress exposure, suggesting that supplementation with GABA could decrease the oxidative stress in Hanwoo steers. However, increased doses of rumen-protected GABA did not tend to promote antioxidant stability during storage (p>0.05).

The rate of lipid oxidation could also be recorded through changes in the myoglobin percentage during storage. The myoglobin percentage represents the degree of meat freshness, which is an important trait for the determination of meat discoloration [7]. The percentage of myoglobin is directly associated with the color formation of the meat, where a larger amount of oxymyoglobin is related to a brighter red color and a brown color representing meat discoloration is caused by a higher percentage of metmyoglobin [36]. This study found that diets supplemented with GABA could lead to better antioxidative stability during cold storage. Significant differences with higher oxymyoglobin percentages and lower myoglobin percentages were recorded between the control group and the treatment groups (p<0.05), where both treatment groups resulted a higher percentage of oxymyoglobin and a lower percentage of metmyoglobin compared to those in the control group from day 3 until day 9. The degree of change in the myoglobin profile was strongly associated with the lipid oxidation rate within the meat [37] since lipid oxidation increases the rate of myoglobin oxidation via reactivity with primary and secondary products generated from unsaturated fatty acids. Supplementation with GABA could therefore promote significantly better antioxidative stability through the inhibition of myoglobin oxidation [7].

Supplementation of GABA in this study had a limited effect on the intramuscular fatty acid content of Hanwoo steers. A significant difference (p<0.05) was observed only for the myristic acid (C14:0) and eicosapentaenoic acid (C20:5n3) contents, where the treatment groups had a higher percentage of the mentioned fatty acid chains than the control group (p<0.05). Additionally, supplementation with GABA seemed to have a significant effect on saturated fatty acid percentages (p<0.05). As demonstrated in the data, the treatment groups had lower saturated fatty acid contents compared to the control group. In addition, MUFA and PUFA levels did not differ among the treatments. The major MUFA was oleic acid (C18:1n9), followed by palmitoleic acid (C16:1n7). The major PUFAs were linoleic acid (C18:2n6), alpha-linoleic acid (C18:3n3), arachidonic acid (C20:4n6), and eicosapentaenoic acid (C20:5n3). Sufficient supplementation of rumen-protected amino acids could increase the formation of beneficial fatty acids [38], although no difference in fatty acid variation was found between the 100 ppm and 200 ppm supplementation groups.

## CONCLUSION

This study revealed that there was an increasing trend of meat quality and slightly animal performance after GABA supplementation. It may also contributed to the improvements of essential antioxidant enzymes and stable regulation of antioxidant activities in the *longissimus lumborum* of Hanwoo steers, as represented by the lower formation of MDA content and the inhibition of myoglobin oxidation during cold storage. Although supplementation with GABA seemed to have a limited effect on the fatty acid profile of the *longissimus lumborum* of Hanwoo steers, it might significantly lower the percentage of saturated fatty acids. Higher doses of GABA may not significantly promote better animal performance and meat quality, suggesting that dietary supplementation with GABA at a dose of 150 mg/kg feed per day is sufficient to improve the meat quality of Hanwoo steers.

## Figures and Tables

**Table 1:** Body weight gain and feed intake of Hanwoo steers fed with rumen protected γ-aminobutyric acid

Variable	Treatments1)			SEM	p-value

	Control	100 ppm	200 ppm		
Animal performance					
Initial weight (kg)	647.43	635.00	657.57	15.79	0.83
Final weight (kg)	695.86	738.14	749.43	18.86	0.45
Weight gain (kg)	63.71^b^	103.14^a^	91.85^ab^	3.45	<0.05
Average daily gain (g/d)	0.65^b^	0.86^a^	0.77^ab^	0.04	<0.05
Daily dry matter intake2)					
Concentrate (kg)	10	10	10	-	-
γ-aminobutyric acid (ppm)	-	100	200	-	-
Straw (kg)	1.5	1.5	1.5	-	-
Total (kg)	11.5	11.5	11.5	-	-

SEM, standard error of the mean; TDN, total digestible nutrient; CP, crude protein.

1) Control, basal diet with 74% TDN and 12% CP; 100 ppm, basal diet supplemented with γ-aminobutyric acid at 100 ppm; 200 ppm, basal diet supplemented with γ-aminobutyric acid at 200 ppm.

2) Feeding was given as *ad libitum* daily.

a,b Means within each row are significantly different (p<0.05).

**Table 2:** Carcass traits of Hanwoo steers fed with rumen protected γ-aminobutyric acid

Variable	Treatments1)			SEM	p-value

	Control	100 ppm	200 ppm		
Yield traits2)					
Hot carcass weight (kg)	442	441.4	460.5	24.51	0.46
Backfat thickness (mm)	73	73.4	80	3.01	0.26
Rib eye area (cm^2^)	10.8	12.4	14.6	1.66	0.27
Yield index3)	4	4	4.5	0.37	0.44
Yield grade4)	61.41	60.5	59.03	1.37	0.41
Quality traits	3	3	3	0	0.41
Marbling score5)	4	4	4	0	0.58
Meat color6)	1.2	1.4	1	0.1	0.35
Fat color7)	2.8	3	3	0.1	0.35
Firmness8)	1.8	1.6	1.4	0.22	0.39
Maturity9)	3	2.8	3.4	0.18	0.35
Quality grade10)	442	441.4	460.5	24.51	0.47

SEM, standard error of the mean; TDN, total digestible nutrient; CP, crude protein.

1) Control, basal diet with 74% TDN and 12% CP; 100 ppm, basal diet supplemented with γ-aminobutyric acid at 100 ppm; 200 ppm, basal diet supplemented with γ-aminobutyric acid at 200 ppm.

2) Area was measured from longissmus thoracis muscle.

3) Yield index was calculated by equation 65.834–[0.393×Backfat thickness (mm)]+[0.088×Ribeye area (cm^2^)]–[0.008×carcass weight (kg)]+2.01.

4) A grade (yield index ≥69.00) = 3; B grade (66.00≤yield index<69.00) = 2; C grade (yield index <66.00) = 1.

5) Marbling score standard; No.1-No.9 (1 = devoid, 9 = abundant).

6) Meat color standard; No.1-No.7 (1 = brightly cherry red, 7 = extremely dark red).

7) Fat color standard; No.1-No.7 (1 = white, 7 = dark yellow).

8) Firmness score; 1 to 3 (1 = soft, 3 = firm).

9) Maturity score; 1 to 3 (1 = youthful, 3 = mature).

10) 1++ grade = 5; 1+ grade = 4; 1 grade = 3; 2 grade = 2; 3 grade = 1.

**Table 3:** Proximate composition (%) and physical properties of *longissimus lumborum* from Hanwoo steers fed with rumen protected γ-aminobutyric acid

Variable	Treatments1)			SEM	p-value

	Control	100 ppm	200 ppm		
Mositure	66.91	65.91	64.1	1.23	0.13
Crude fat	13.57	12.89	14.25	1.16	0.78
Crude protein	16.27^b^	18.28^a^	19.19^a^	0.26	<0.05
Crude ash	0.49	0.49	0.47	0.01	0.42
Water holding capacity (%)	74.86^b^	75.38^b^	81.31^a^	0.72	<0.05
Cooking loss (%)	29.65^a^	29.01^a^	25.52^b^	0.22	<0.05
Shear force (kg)	5.71	4.92	4.91	0.23	0.06

SEM, standard error of the mean; TDN, total digestible nutrient; CP, crude protein.

1) Control, basal diet with 74% TDN and 12% CP; 100 ppm, basal diet supplemented with γ-aminobutyric acid at 100 ppm; 200 ppm, basal diet supplemented with γ-aminobutyric acid at 200 ppm.

a,b Means within each row are significantly different (p<0.05).

**Table 4:** Effect of rumen protected γ-aminobutyric acid supplementation on antioxidant enzymes of *longissimus lumborum* from Hanwoo steers during refrigerated storage

Variable	Storage (d)	Treatments1)			SEM	p-value		
	
		Control	100 ppm	200 ppm		Diet	Storage	Diet×storage
CAT (unit/g meat)	0	321.5^c^,^x^	341.3^ab^,^x^	350.1^a^,^x^	3.13	<0.05	<0.001	<0.05
	3	318.4^b^,^x^	337.2^ab^,^x^	347^a^,^x^	4.02			
	6	315.2^b^,^y^	334.1^ab^,^y^	343.9^a^,^y^	4.04			
	9	309.1^b^,^y^	327.8^ab^,^y^	337.6^a^,^y^	5.05			
SOD (unit/g meat)	0	51.9^b^,^x^	53.3^a^,^x^	53.1^a^,^x^	0.01	<0.05	<0.05	<0.05
	3	50^b^,^x^	52.5^a^,^x^	52.4^a^,^x^	0.014			
	6	48.2^b^,^x^	51.8^a^,^x^	51.6^a^,^y^	0.01			
	9	43.2^b^,^y^	50.9^a^,^y^	51^a^,^y^	0.01			
GSH-Px (unit/g meat)	0	1.62^x^	1.68^x^	1.7^x^	0.02	0.07	<0.05	<0.05
	3	1.51^y^	1.59^y^	1.59^x^	0.00			
	6	1.36^y^	1.4^y^	1.42^y^	0.03			
	9	1.05^z^	1.14^z^	1.21^z^	0.01			

SEM, standard error of the mean; CAT, catalase; SOD, superoxide dismutas; GSH-Px, glutathione peroxidase; TDN, total digestible nutrient; CP, crude protein.

1) Control, basal diet with 74% TDN and 12% CP; 100 ppm, basal diet supplemented with γ-aminobutyric acid at 100 ppm; 200 ppm, basal diet supplemented with γ-aminobutyric acid at 200 ppm.

a–c Means within each row are significantly different (p<0.05).

xy Means within each column are significantly different (p<0.05).

**Table 5:** Effect rumen protected γ-aminobutyric acid supplementation on pH and 2-thiobarbituric acid reactive substances level (mg malondialdehyde/kg meat) of *longissimus lumborum* from Hanwoo steers during refrigerated storage

Variable	Storage (d)	Treatments1)			SEM	p-value		
	
		Control	100 ppm	200 ppm		Diet	Storage	Diet×storage
pH	0	5.34	5.36	5.34	0.07	0.098	<0.001	0.05
	3	5.35	5.37	5.38	0.09			
	6	5.35	5.39	5.39	0.01			
	9	5.57	5.61	5.61	0.03			
TBARS	0	0.32	0.32	0.28	0.02	<0.05	<0.05	0.43
	3	0.43^a^	0.4^a^	0.35^b^	0.01			
	6	0.49^a^	0.43^ab^	0.39^b^	0.03			
	9	0.6^a^	0.45^b^	0.39^c^	0.03			

SEM, standard error of the means; TBARS, 2-thiobarbituric acid reactive substances; TDN, total digestible nutrient; CP, crude protein.

1) Control, basal diet with 74% TDN and 12% CP; 100 ppm, basal diet supplemented with γ-aminobutyric acid at 100 ppm; 200 ppm, basal diet supplemented with γ-aminobutyric acid at 200 ppm.

a–c Means within each row are significantly different (p<0.05).

**Table 6:** Effect of rumen protected γ-aminobutyric acid supplementation on meat color and relative myoglobin concentration (%) of *longissimus lumborum* from Hanwoo steers during refrigerated storage

Variable	Storage (d)	Treatments1)			SEM	p-value		
	
		Control	100 ppm	200 ppm		Diet	Storage	Diet×storage
CIE L^*^	0	34.63	33.86	34.72	0.92	0.09	0.75	0.55
	3	32.10	32.94	33.72	0.67			
	6	33.84	33.56	34.97	0.84			
	9	34.02	35.88	35.74	0.25			
CIE a^*^	0	22.37	21.42	22.44	0.45	<0.05	<0.05	0.27
	3	20.56	20.99	21.10	0.89			
	6	22.87	22.55	23.18	0.34			
	9	13.62^b^	16.00^a^	16.73^a^	0.26			
CIE b^*^	0	12.34	12.38	12.85	0.34	0.65	<0.001	0.43
	3	11.17	11.51	11.89	0.28			
	6	10.87	11.47	11.74	0.15			
	9	9.08	9.42	8.88	0.75			
Oxymyoglobin (%)	0	59.38	60.47	61.37	1.14	<0.05	<0.001	0.47
	3	45.21^b^	49.91^a^	50.22^a^	1.29			
	6	31.71^b^	39.51^a^	39.75^a^	1.25			
	9	15.39^b^	24.27^a^	26.28^a^	2.65			
Deoxymyoglobin (%)	0	26.23	27.59	27.45	1.36	0.959	<0.05	0.44
	3	26.36	26.03	27.27	0.38			
	6	19.35	17.58	18.21	0.99			
	9	18.25	18.69	18.66	0.43			
Metmyoglobin (%)	0	22.95	21.96	21.5	0.73	<0.05	<0.001	0.75
	3	38.47^a^	32.84^b^	32.17^b^	0.19			
	6	34.71	32.98	33.1	0.21			
	9	52.61^a^	45.73^b^	47.85^b^	1.09			

SEM, standard error of the means; CIE, Commission Internationale de l’Eclairage; TDN, total digestible nutrient; CP, crude protein.

1) Control, basal diet with 74% TDN and 12% CP; 100 ppm, basal diet supplemented with γ-aminobutyric acid at 100 ppm; 200 ppm, basal diet supplemented with γ-aminobutyric acid at 200 ppm.

a,b Means within each row are significantly different (p<0.05).

**Table 7:** Effect of rumen protected γ-aminobutyric acid supplementation on total fatty acid concentration (%) of Hanwoo steers

Items	Treatments1)			SEM	p-value

	Control	100 ppm	200 ppm		
C14:0	3.76^a^	3.25^ab^	3.16^b^	0.15	<0.05
C16:0	30.40	28.33	26.97	0.46	0.79
C16:1n7	5.81	5.29	5.97	0.20	0.85
C18:0	10.12	10.37	8.76	0.29	0.87
C18:1n9	46.75	49.53	52.08	0.58	1.00
C18:2n6	2.82	2.79	2.76	0.12	0.20
C18:3n3	0.25	0.26	0.27	0.02	0.18
C20:4n6	0.05	0.05	0.03	0.00	0.87
C20:5n3	0.04^a^	0.04^a^	0.02^b^	0.00	<0.01
SFA	44.28^a^	43.05^a^	38.89^b^	0.53	<0.05
MUFA	52.57	54.82	58.05	0.53	0.94
PUFA	3.16	3.13	3.07	0.14	0.21
n-3	2.86	2.84	2.78	0.02	0.44
n-6	0.29	0.29	0.29	0.12	0.20
n-6/n-3	10.55	10.24	9.98	0.54	0.72

SEM, standard error of the means; SFA, saturated fatty acid; MUFA, mono unsaturated fatty acid; PUFA, poly unsaturated fatty acid; TDN, total digestible nutrient; CP, crude protein.

1) Control, basal diet with 74% TDN and 12% CP; 100 ppm, basal diet supplemented with γ-aminobutyric acid at 100 ppm; 200 ppm, basal diet supplemented with γ-aminobutyric acid at 200 ppm.

a,b Means within each row are significantly different (p<0.05).
